# Ensemble Machine Learning Approach Improves Predicted Spatial Variation of Surface Soil Organic Carbon Stocks in Data-Limited Northern Circumpolar Region

**DOI:** 10.3389/fdata.2020.528441

**Published:** 2020-10-28

**Authors:** Umakant Mishra, Sagar Gautam, William J. Riley, Forrest M. Hoffman

**Affiliations:** ^1^ Bioscience Division, Sandia National Laboratory, Livermore, CA, United States; ^2^ Earth and Environmental Sciences, Lawrence Berkeley National Lab, Berkeley, CA, United States; ^3^ Climate Change Institute, Oak Ridge National Laboratory, Oak Ridge, TN, United States

**Keywords:** soil organic carbon, spatial prediction, machine learning, permafrost soils, environmental controllers

## Abstract

Various approaches of differing mathematical complexities are being applied for spatial prediction of soil properties. Regression kriging is a widely used hybrid approach of spatial variation that combines correlation between soil properties and environmental factors with spatial autocorrelation between soil observations. In this study, we compared four machine learning approaches (gradient boosting machine, multinarrative adaptive regression spline, random forest, and support vector machine) with regression kriging to predict the spatial variation of surface (0–30 cm) soil organic carbon (SOC) stocks at 250-m spatial resolution across the northern circumpolar permafrost region. We combined 2,374 soil profile observations (calibration datasets) with georeferenced datasets of environmental factors (climate, topography, land cover, bedrock geology, and soil types) to predict the spatial variation of surface SOC stocks. We evaluated the prediction accuracy at randomly selected sites (validation datasets) across the study area. We found that different techniques inferred different numbers of environmental factors and their relative importance for prediction of SOC stocks. Regression kriging produced lower prediction errors in comparison to multinarrative adaptive regression spline and support vector machine, and comparable prediction accuracy to gradient boosting machine and random forest. However, the ensemble median prediction of SOC stocks obtained from all four machine learning techniques showed highest prediction accuracy. Although the use of different approaches in spatial prediction of soil properties will depend on the availability of soil and environmental datasets and computational resources, we conclude that the ensemble median prediction obtained from multiple machine learning approaches provides greater spatial details and produces the highest prediction accuracy. Thus an ensemble prediction approach can be a better choice than any single prediction technique for predicting the spatial variation of SOC stocks.

## Introduction

High latitude permafrost region soils store large stocks of soil organic carbon (SOC) due to multiple cryopedogenic processes operating over long time scales (Ping et al., 2008; Tarnocai et al., 2009; Hugelius et al., 2014; Ping et al., 2015). Enhanced rate of climate warming at high latitudes is causing widespread degradation and thawing of permafrost soils and subsequent release of greenhouse gases such as CO_2_ and CH_4_ to the atmosphere (Romanovsky et al., 2010; Rowland et al., 2010; Biskaborn et al., 2019). As a significant portion of permafrost region SOC stocks has the potential to be emitted as greenhouse gases under changing climate (McGuire et al., 2016; McGuire et al., 2017), permafrost region SOC stocks are a vulnerable component of the global carbon cycle. Current earth system models show large uncertainty both in baseline SOC stock representations and their release to the atmosphere under changing climate (Mishra et al., 2013; Schuur et al., 2015; McGuire et al., 2016). Reliable estimates of the magnitude and spatial variation of permafrost region SOC stocks are essential to better understand the environmental controls and to reduce the uncertainty in predicting permafrost region carbon -climate feedbacks. The magnitude of SOC stored in the soil per unit of land area is highly variable in permafrost region soils (Mishra and Riley, 2015; Mishra et al., 2017), as SOC stocks depend on various environmental factors such as soil type, land use, topographic features, and climatic conditions, which are site specific. Knowledge of soil and site-specific environmental controllers is essential to make reliable spatial predictions of SOC stocks.

In spatial prediction of soil properties, mathematical or statistical relationships are usually developed using limited number of soil observations and environmental predictors. The derived relationship is then applied with environmental predictors across the study area to produce spatially-explicit estimates of soil properties. A number of spatial prediction approaches have been used to predict the spatial variation of SOC stocks depending upon the available data density and environmental data of soil-forming factors (Mishra and Lal, 2010; Minasny et al., 2013). Spatial prediction techniques can broadly be categorized into three groups that use: 1) environmental correlation between soil C and environmental factors (Martin et al., 2011; Zhang et al., 2011); 2) spatial autocorrelation among soil C observations (Mishra et al., 2009; Cambule et al., 2013); and 3) hybrid approaches that combine environmental correlation and spatial autocorrelation (Martin et al., 2014; Meng, 2014). Among spatial prediction approaches used to predict the spatial variation of SOC stocks, multiple linear regressions (group 1 that uses environmental correlation) and ordinary kriging (group 2 that uses spatial autocorrelation) are the most commonly used techniques in the literature, primarily because of their simplicity in interpretation and ease of use. However, the most accurate predictions (lowest prediction errors) have been achieved through the use of hybrid approaches [e.g., regression kriging (Hengl et al., 2007; Minasny et al., 2013; group 3] that combined environmental correlation and spatial autocorrelation.

In addition to the above-mentioned three groups of spatial prediction, methods with increasing computational complexity are being used to predict the spatial variation of soil properties. For example, machine-learning based spatial modeling techniques such as random forest (Sreenivas et al. 2016; Siewert 2018), neural networks (Li et al., 2013), and rule-based models (Viscarra Rossel and Webster, 2012; Lacoste et al., 2014) have been used to capture non-linear relationships between soil C and environmental factors. These machine learning approaches are being increasingly applied for predicting soil properties including SOC stocks. More recently, ensembles of multiple approaches are also being applied to improve the spatial prediction of SOC stocks (Vasat et al., 2017; Chen et al., 2020). The use of average or median predictions from ensemble of different approaches improves spatial prediction of soil properties and the inter quartile range of ensemble predictions provides estimates of uncertainty ranges due to different model structures (McGuire et al., 2016; McGuire et al., 2017; Shi et al., 2018). Further, the spatial distribution of uncertainty estimates can also inform future sampling locations to reduce the existing uncertainty.

Permafrost affected soils show vast spatial and vertical heterogeneity of soil properties (Johnson et al., 2011; Siewert et al., 2015; Beer, 2016), and therefore areal estimates of permafrost region soil properties, including SOC stocks, could benefit from advanced spatial modeling approaches. However, application of geospatial approaches in the permafrost region has been limited due to low sample density and limited availability of spatially resolved environmental datasets (Mishra et al., 2013; Siewert, 2018). Recently, spatial predictions of soil properties using geospatial and remote sensing information have been applied at local to regional scales to account for and better represent the spatial variation of permafrost affected soil properties (Pastick et al., 2014; Bartsch et al., 2016; Ding et al., 2016; Siewert, 2018). These high-resolution predictions using a variety of geospatial techniques have demonstrated promising results in the permafrost terrain.

Multiple studies have documented that the regression kriging approach produces lower prediction errors (Hengl et al., 2007; Kumar et al., 2012; Meng, 2014) in comparison to other spatial prediction approaches. We designed this study to compare the prediction accuracy of regression kriging with different machine learning approaches. We hypothesized that because regression kriging approach captures both spatial autocorrelation and environmental correlation, it will produce lower prediction errors in comparison to machine learning approaches, which capture mainly non-linear relations between soil properties and environmental factors. The specific objectives in this study are to 1) compare prediction accuracy of machine learning approaches with regression kriging, 2) determine the importance of environmental predictors across different spatial prediction approaches, 3) evaluate the accuracy of individual and combined (ensemble) ML approaches, and (4) create a high-resolution estimate of surface (0–30 cm) northern circumpolar region SOC stocks using an ensemble machine learning approach.

## Materials and Methods

### Spatial Variation in Environmental Factors of the Permafrost Region

A digital elevation model with 250-m spatial resolution was obtained from the US Geological Survey (Danielson and Gesch, 2011). Elevations ranged from sea level to 6,130 m in the northern circumpolar region. The digital elevation model was used to calculate seven major topographic attributes (elevation, slope, aspect, flow accumulation, topographic wetness index, sediment transport index, and stream power index) to evaluate their use in predicting the spatial variation of surface SOC stocks. Average annual (1960–1990) precipitation and temperature data at 1-km spatial resolution were obtained from the global climate data of Hijmans et al. (2005). This interpolated dataset was generated for global land surfaces using latitude, longitude and elevation as independent variables. In the northern circumpolar region, average annual precipitation ranged from 52 mm in the Russian Arctic Desert to 2,956 mm in southeast Greenland. Average annual temperatures were lowest in northern Canada and Greenland (−28° to −20°C) and highest in southern Canada (3° to –10°C).

Global land cover data at 250-m spatial resolution were obtained from the European Space Agency (Glob cover, 2009). Of the total land area in the northern circumpolar region, Needleleaf mixed forest covered 31%, sparse vegetation covered 27%, permanent snow and ice covered 11%, and shrub land covered 7%. The bedrock geology data was obtained from the global lithological map produced by Hartmann and Moosdorf (2012). In the northern circumpolar region, the largest proportion of land area had mixed sedimentary rocks (24.7%), followed by siliciclastic sedimentary rocks (19%), metamorphic rocks (12.4%), and ice and glaciers (12.1%). The smallest proportions of land area were underlain by evaporates (0.05%), pyroclastics (0.37%), and acid volcanic rocks (0.73%). For this study, we resampled all the environmental data into a common spatial resolution of 250 m. Continuous environmental variables were resampled using bilinear interpolation and categorical variables were resampled using nearest neighbor resampling technique by using the resample function of ArcGIS (ArcGIS version 10.4, Environmental Systems Research Institute, Inc., Redlands, CA, United States). The soil type information of the study area was obtained from the soil order map of Tarnocai et al. (2009). The largest soil area in the study domain was under Gelisols (57%), followed by Histosols (5%), and remaining mineral soils Spodosols, Inceptisols, Mollisols, Entisols, Alfisols, Andisols, and Aridisols covered 38% soil area.

### Soil Organic Carbon Profile Observations and Their Distribution Across Environmental Factors

**FIGURE 1:**
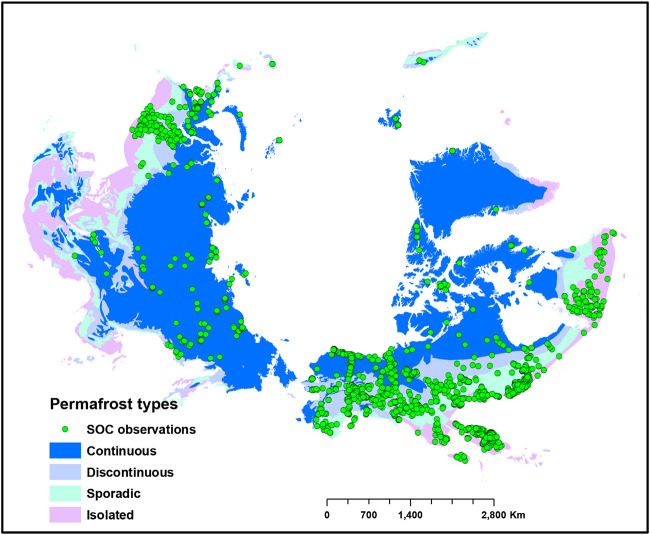
Study area and distribution of soil samples (*n* = 2,374). Green dots show distribution of soil organic carbon (SOC) observations across different permafrost types of the Northern circumpolar permafrost region.

We compiled and updated the existing SOC data for permafrost affected soils from various sources. In addition to the SOC data used by Michaelson et al. (2013), Hugelius et al. (2014), Palmtag et al. (2015), Siewert et al. (2015), and Vitharana et al. (2017), we collected additional georeferenced SOC profile observations from individual investigators from Canada, Russia, South Korea, and Sweden. Figure 1 shows the spatial distribution of SOC profile observations across the study area.

**FIGURE 2:**
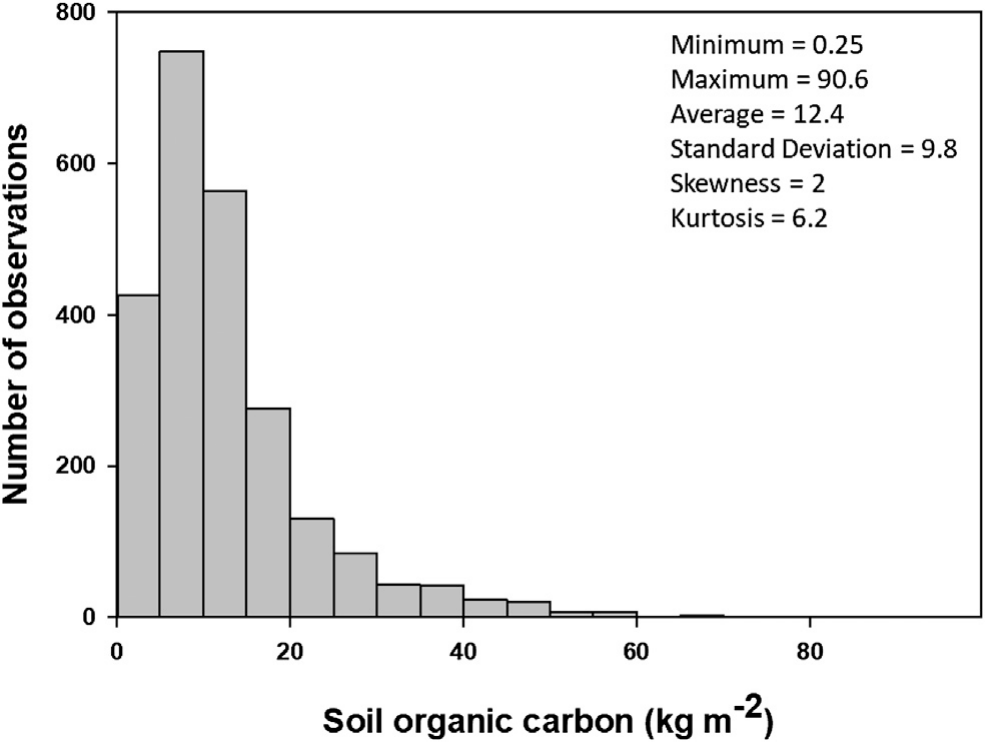
Histogram and descriptive statistics of surface soil organic carbon stocks (0–30 cm) observations used in this study (*n* = 2,374).

The collected soil observations are broadly representative of the heterogeneity of environmental conditions of the northern circumpolar region permafrost affected soils. The SOC profile observations represented 13 different land cover types. The largest number of samples were from the Needleleaf forest land cover type (34.3%), followed by sparse vegetation (25.5%), and mosaic forest shrubland vegetation (10.5%). The smallest number of samples were from broadleaf deciduous forest (0.12%), followed by broadleaf evergreen forest (0.17%), and shrublands (0.21%). The SOC observations captured a large range of climatic factors: mean annual precipitation ranged from 820 to 1,625 mm and mean annual temperature ranged from −20° to 6°C. The SOC observations were distributed from sea level to 3,000 m in elevation and captured a large range of slope angles (0.5 to 44.5°). SOC samples covered 11 of 14 bedrock geology types in the circumpolar region. The largest number of samples were from siliciclastic sedimentary rocks (34.5%), mixed sedimentary rocks (25.3%), and unconsolidated sediments (8.7%). The smallest number of samples were from basic plutonic rocks (0.25%), intermediate plutonic rocks (0.3%), and acid volcanic rocks (1%).

### Spatial Prediction of SOC Stocks

#### Regression Kriging

Regression Kriging is a widely used spatial interpolation technique in soil science, which combines a linear regression of dependent variable such as SOC stocks with environmental variables with kriging of the regression residuals (Hengl et al., 2007; Keskin and grunwald, 2018; Wu et al., 2019). In this method, the SOC stocks at an unsampled location are predicted by adding the interpolated regression residuals into the regression predicted SOC stocks. This approach can be summarized by:

zLRK^(s0 )=mMLR^(s0)+eOK^(s0)

Where, zLRK^(s0) is the estimated SOC stocks at location$\;s_{0}$, mMLR^is the value predicted from multiple linear regressions (MLR), and eOK^ is the kriged values of the MLR residuals at point $S_{0}$ using ordinary kriging. In summary, in this study the forward stepwise multiple linear regression was used to identify the statistically significant predictors of SOC stocks of the study area. Then Ln-transformed SOC stocks model residuals were calculated for the sample locations and covariance structure of the model residuals was fitted using a variogram model. Regression residuals were then interpolated using ordinary kriging and added to the estimated Ln-transformed SOC stocks regression model surface (Mishra et al., 2012). Several recent studies have also applied a variant of this technique using a geographically weighted regression approach to model the spatially varying regression relationships between the SOC stocks and its environmental controllers (Zhang et al., 2011; Mishra and Riley, 2015; Mitran et al., 2018).

#### Machine Learning Approaches

Machine learning approach is a family of algorithms which do not assume any mechanistic nature to the data and instead seek to “learn” a function that best maps input parameters to an output. We used gradient boosting machine (GBM), multinarrative adaptive regression spline (MARS), support vector machine (SVM), and random forests (RF) machine learning approaches to predict the surface SOC stocks which were previously used to predict soil properties in a variety of environments. Individual predictions from these machine learning techniques and their ensemble median were compared with the SOC stocks predicted by the regression kriging approach. The GBM algorithm which was originally proposed by Friedman (2001), uses simple regression model “weak learners” and iteratively combine this simple model to obtain “strong learner” with improved accuracy by reducing the bias and the variance. GBM model include two major user defined parameters; number of tree and tree depth. The tree depth of 3 and number of trees of 150 were used based on the minimum root mean squared error (RMSE) of prediction. MARS, which was introduced by Friedman (1991), computes the underlying nonlinear patterns hidden in the data. It builds the relationship between the response and dependent variable using distinct set of coefficients and the function which are controlled by the regression. MARS optimization is a two-step process. In the first step, a large number of basis functions (connected splines) are constructed to overfit the data and in the second step the basis functions are selected based on best fit. The tuning parameters for MARS include the nprune and degree. The nprune value of 18 and degree value of 1 were used based on the minimum RMSE of prediction. The SVM, originally proposed by Cortes and Vapnik (1995), sets up a decision boundary in the feature space to separate different classes. Mathematically, it creates best fit hyperplanes between the classes to minimize errors. The objective function intends to select the best hyperplane with largest margin between the classes, where margin is the sum of distance between the separating hyperplane and nearest points of different class in either side of the hyperplane. The tuning parameters for SVM includes sigma and C. The sigma value of 0.14 and C value of 1 were used based on the minimum RMSE of prediction. RF is a tree-based machine learning approach that works by building a set of regression trees and averaging the results for final prediction (Breiman, 2001). RF works on a rationale that the combination of learning models (tree-based ensemble) increases the prediction accuracy. It consists of an ensemble of randomized classification and regression trees (CART, Breiman et al., 1984), where many decision trees are built using a random subsample of the available environmental factors. The final result is a single prediction constructed as a weighted average over all these individually suboptimal trees. In the RF approach, the model parameters that needed specification were: 1) the number of trees to grow in the forest (ntree = 500), 2) the number of randomly selected predictor variables at each node (mtry = 5), and 3) the minimum number of observations at the terminal nodes of the trees (nodesize = 5). RF has been reported to have high predictive performance, low correlation of individual trees, and small bias and provides information on the relative importance of predictors (Breiman, 2001; Svetnik et al., 2003; Arun and Langmead, 2005).

### Evaluation of Prediction Accuracy of Different Geospatial Approaches

We calculated four validation indices that demonstrate different quality attributes of predicted SOC stock maps: 1) the measure of inaccuracy expressed as the RMSE, 2) the measure of bias expressed as the mean estimation error (MEE), 3) the measure of linear relationship between observed and predicted SOC stock values expressed as Pearsons’s correlation coefficient (r), and 4) the ratio of performance to deviation (RPD), which is the ratio of the standard deviation of the observed SOC stock values to the RMSE of the predictions. The larger the RPD, the more accurate the prediction. To calculate these validation indices, we split the SOC stock dataset into four different spatially balanced calibration and validation datasets (70/30, 75/25, 85/15, 90/10) using “create subset” function of ArcGIS (ArcGIS version 10, Environmental Systems Research Institute, Inc., Redlands, CA, United States). We reported average validation indices obtained from these four validation subsets. The predicted SOC stock values from all the prediction approaches were extracted at validation data sites and the following equations were applied:

$$MEE=\;\frac{1}{n}\overset{n}{\underset{i=1}{\sum }}\left[S\hat{O}C\left(x_{i}\right)-\;SOC\left(x_{i}\right)\right]$$

$$RMSE=\;\sqrt{\frac{1}{n}\overset{n}{\underset{i=1}{\sum }}{\left[S\hat{O}C\left(x_{i}\right)-\;SOC\left(x_{i}\right)\right]}^{2}}$$

where $SOC\left(x_{i}\right)$ is the measured SOC, $S\hat{O}C\left(x_{i}\right)$ the estimated SOC, and *n* is the number of validation observations (*n* = 714). For optimal predictions, MEE and RMSE values should approach zero. Chang and Laird (2002) defined three classes of RPD; models that have high predictive ability (RPD > 2), models that have intermediate predictive ability that can be possibly improved (RPD between 1.4 and 2), and models that have no predictive ability (RPD < 1.4). In addition to predict the spatial variation of SOC stocks at 250-m spatial resolution across the study area, we also calculated the coefficient of variation of surface SOC stocks across different permafrost zones of the Northen circumpolar permafrost region.

## Results

### Descriptive Statistics of Soil Organic Carbon Observations

**FIGURE 3:**
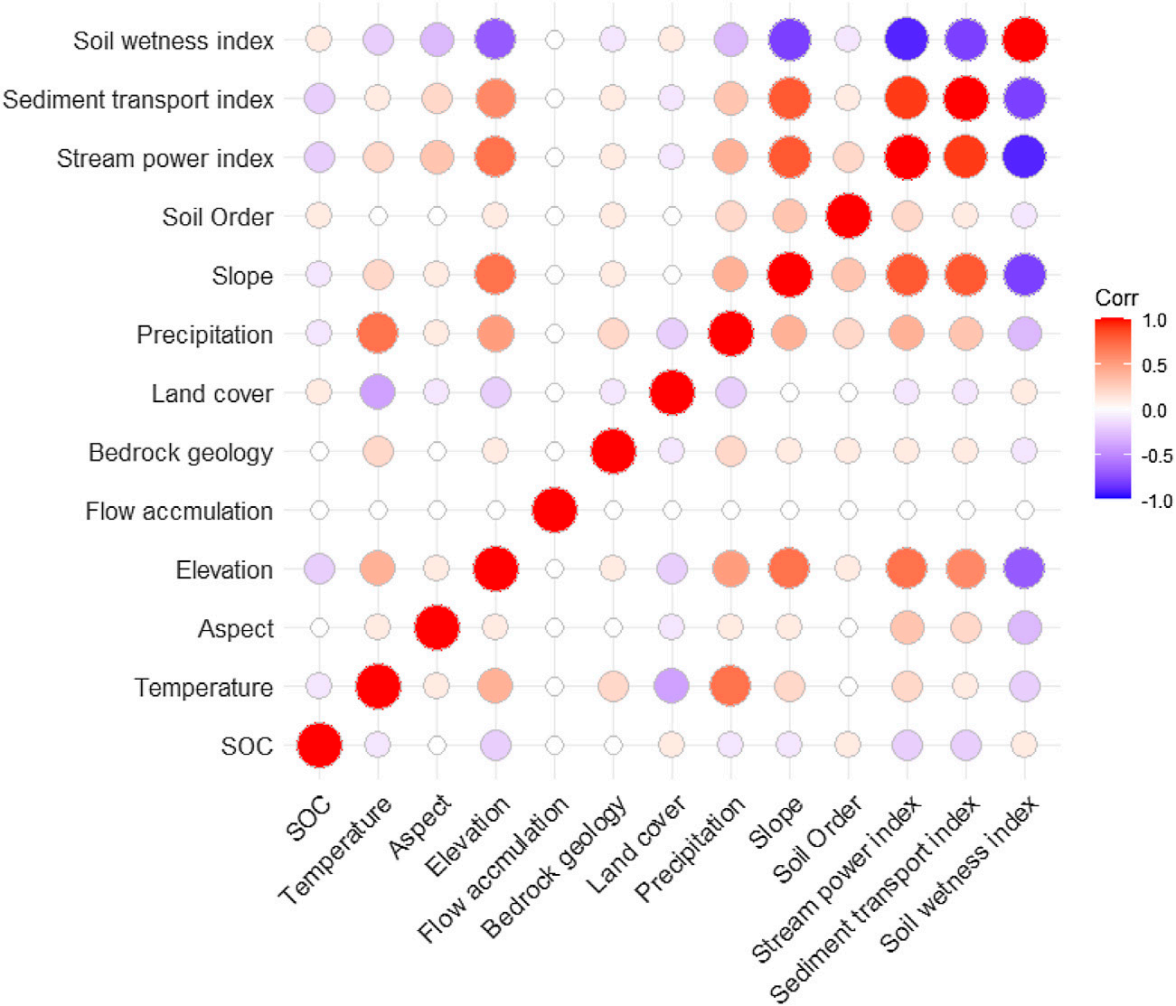
Pearson’s correlation coefficients between SOC stocks and environmental variables used in this study. The insignificant correlations (*p* value >0.05) are blank (white).

Statistical properties of the surface SOC profile observations at calibration and validation sites are summarized in Figure 2. The average surface SOC stock of northern circumpolar region was 12.5 kg m^−2^, ranging from 0.25–90 kg m^−2^. The observed SOC stocks showed unimodal (kurtosis = 2) and positively skewed (coefficient of skewness = 2.1) distributions. Among total SOC observations, 2% of the samples had SOC stocks less than 1 kg m^−2^, and about 6% of the samples had SOC stocks larger than 30 kg m^−2^. The majority of samples (92%) had SOC stocks between 1 and 30 kg m^−2^. The SOC stock values of validation samples were within the range of calibration samples. Figure 3 shows the linear relations between SOC stocks and different environmental factors used in this study.

### Predicted Spatial Variation of Surface Soil Organic Carbon Stocks

**FIGURE 4:**
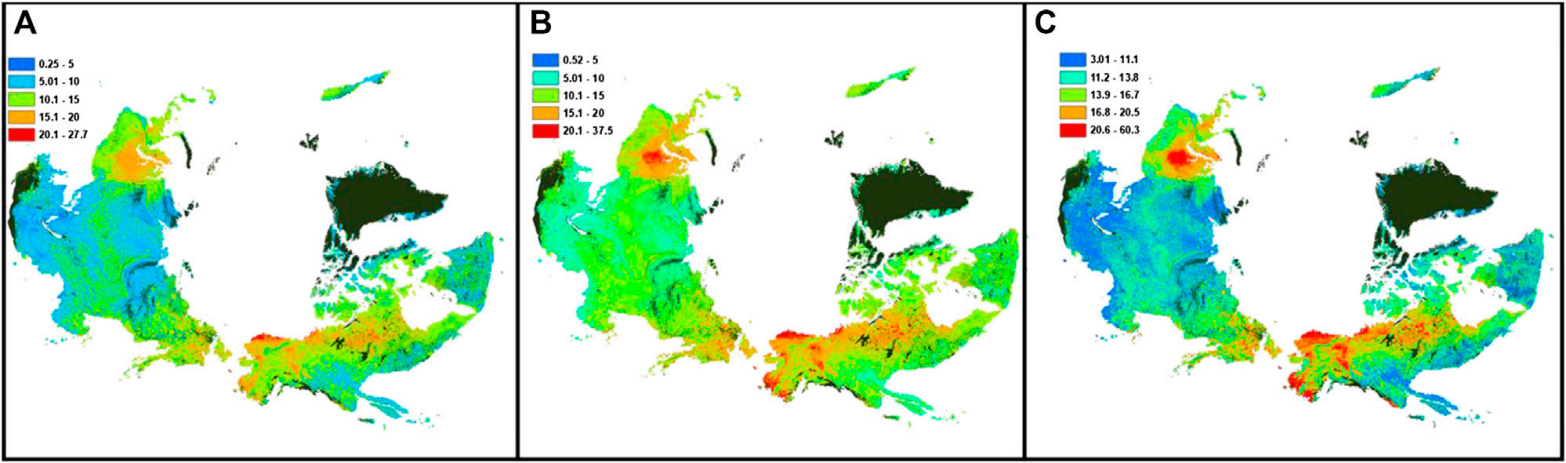
Predicted spatial variation of surface (0–30 cm) SOC stocks (median predictions; **(B)** of the northern circumpolar permafrost region using ensemble machine learning approach, with its lower quartile **(A)** and upper quartile **(C)**. Areas in black show water surface or perennial ice, urban, and barren land with consolidated materials.

Due to lowest prediction errors of surface SOC stocks obtained, we used the results of the ensemble median of the machine learning approaches to describe the magnitude and spatial variation of surface SOC stocks (Figure 4). Predicted median surface SOC content showed moderate spatial variation (CV = 26%), ranging from 0.5 to 37.5 kg m^−2^, with an average circumpolar region surface SOC content of 12.3 kg m^−2^. Among different permafrost regions, the discontinuous permafrost region showed highest SOC content (12 kg m^−2^, with lower and upper quartiles of 11.0 and 13.5 kg m^−2^, respectively), followed by the sporadic permafrost region (10.5 kg m^−2^, with lower and upper quartiles of 9.0 and 12.0 kg m^−2^, respectively), and the continuous permafrost region (10.0 kg m^−2^, with lower and upper quartiles of 8.6 and 11.0 kg m^−2^, respectively). Lowest surface SOC content was found in isolated permafrost region soils (9.0 kg m^−2^, with lower and upper quartiles of 8.0 and 10.0 kg m^−2^, respectively).

The ensemble median of machine learning approaches predicted total SOC stock $218_{-26}^{+22}$ Pg C in 0–30 cm depth of the northern circumpolar region. Out of this total, the continuous permafrost region contained 54.5% ($119_{-14}^{+12}$ Pg C), the discontinuous permafrost region contained 18% ($39_{-4}^{+4}$ Pg C), sporadic permafrost region contained 14% ($31_{-4}^{+3}$ Pg C), and the isolated permafrost region contained 13% ($29_{-3}^{+4}$ Pg C) of the total surface SOC stocks. The largest spatial variation in predicted surface SOC stocks was found in continuous permafrost region soils (CV = 61%), followed by isolated permafrost region (CV = 50%). Both sporadic and discontinuous permafrost region soils showed similar spatial variation (CV = 39%) in the surface SOC stocks (Table 1).

**TABLE 1:** Average SOC content and predicted total SOC stocks in different permafrost zones within the circumpolar permafrost region.

Permafrost types	Average SOC content (kg m^−2^)	Coefficient of variation (%)	Total SOC stock (Pg C)
Continuous	10.0 (8.6–11)	61.0	119.0 (105–131)
Discontinuous	12.0 (11–13.5)	39.0	39.0 (35–43)
Sporadic	10.5 (9–12)	39.0	31.0 (27–34)
Isolated	9.0 (8–10)	50.0	29.0 (25–32)

Values in parentheses are the lower and upper quartiles.

**FIGURE 5:**
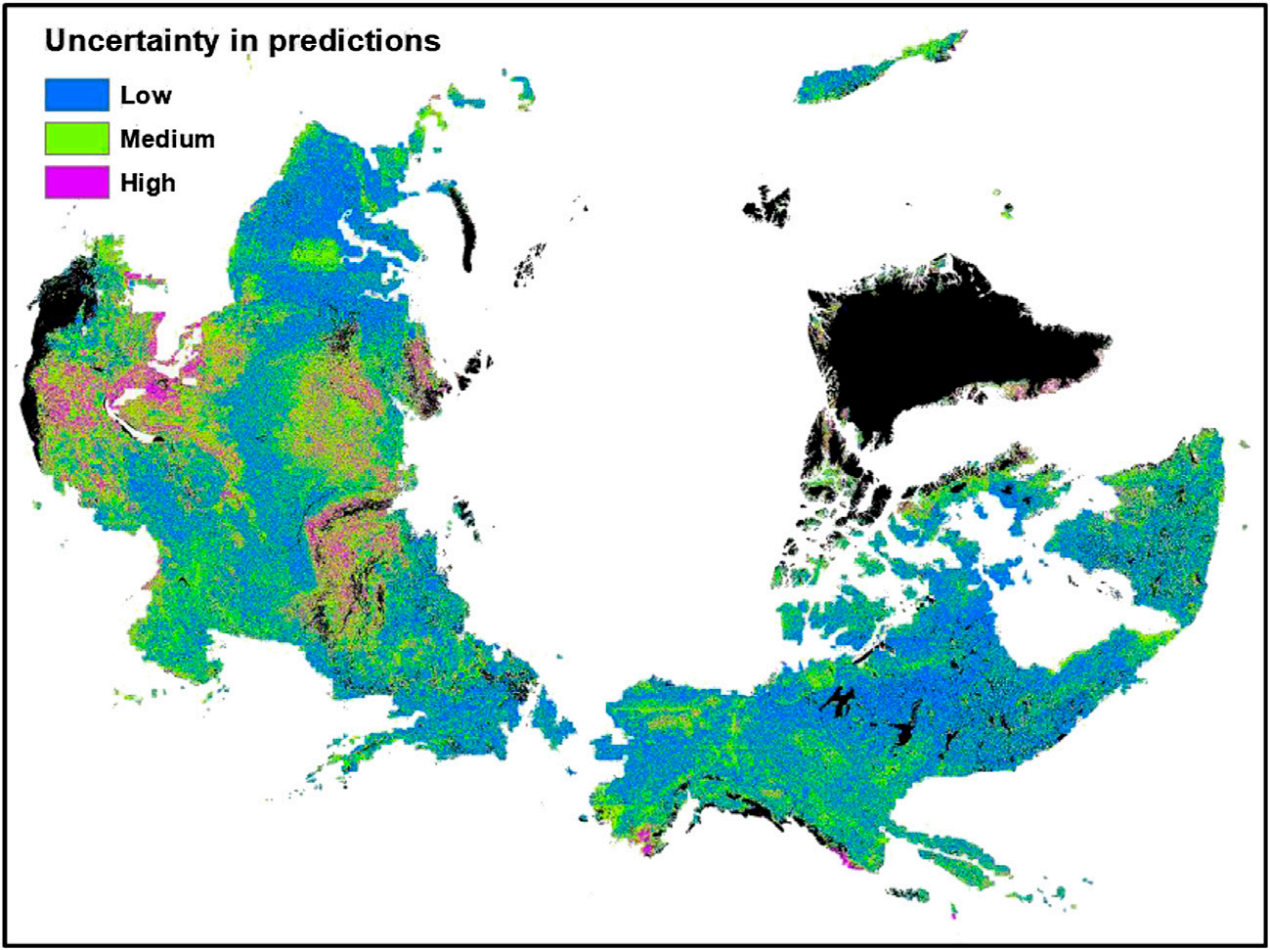
Distribution of uncertainties in the predicted surface soil organic carbon stocks of the northern circumpolar permafrost region. Blue color shows areas with <20% uncertainty, green color shows areas with 20–49% uncertainty and purple color shows areas with >50% uncertainty in the predicted SOC stocks. Areas in black color show water surface or perennial ice, urban, and barren land with consolidated materials.

In general, we observed an inverse spatial relationship between the magnitude of SOC stocks and its uncertainty (expressed as a percent calculated using the lower and upper quartile values), i.e., the areas that stored more SOC stocks (Figure 4) were least uncertain and the areas that stored less SOC stocks were most uncertain. The uncertainty in surface SOC stocks was less than 20% in about half of the study area, shown by blue color in Figure 5. Areas with high uncertainty (>50% uncertainty; purple color in Figure 5) in predicted SOC stocks was observed in small patches in Southern Alaska and Iceland, and in larger areas of Southern and Western Russian permafrost region. Our results showed that 7% of the total study area had high uncertainty in surface SOC stocks. Areas with intermediate uncertainty (20–49% uncertainty; shown by green color in Figure 5) in surface SOC stocks covered about 43% of the study area.

### Environmental Predictors of Surface Soil Organic Carbon Stocks

**FIGURE 6:**
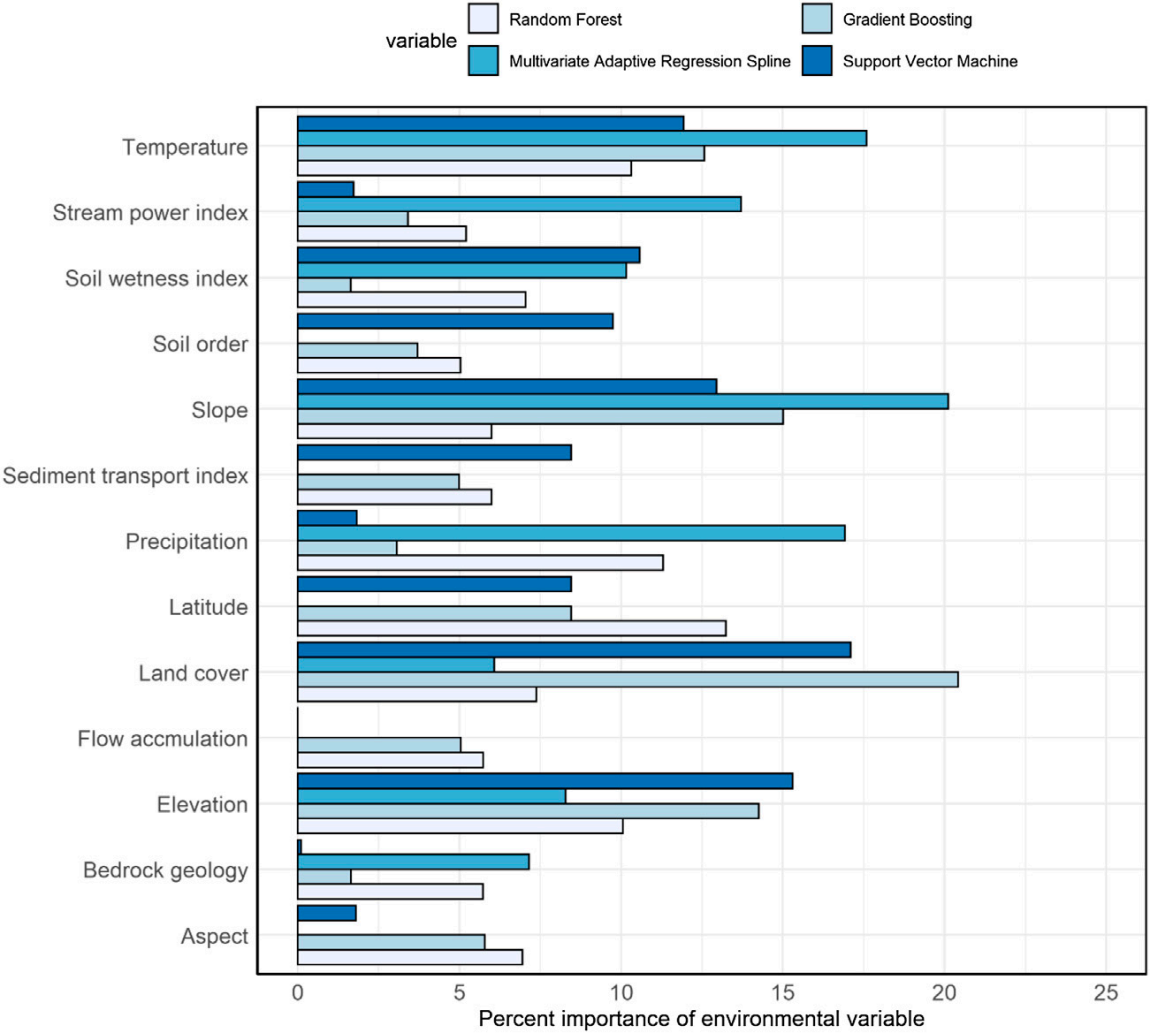
The importance of different environmental predictors differs in different machine learning approaches. Number in the horizontal bars shows the relative importance of environmental predictors of soil organic carbon stocks expressed as percent.

We applied multiple environmental predictors in different prediction approaches, and the number and importance of environmental predictors differed among prediction approaches (Figure 6). In machine learning approaches where we applied all environmental predictors, temperature, land cover types, slope, and elevation had higher impacts and soil types, bedrock geology types, aspect, and sediment transport index had lower impacts on the predicted variation of SOC stocks. In the RF approach, which produced the highest prediction accuracy (lowest RMSE) among machine learning approaches, average annual temperature and precipitation, latitude, and elevation were the most important environmental predictors of surface SOC stocks. Similarly, soil types, bedrock geology types, and surface hydrology attributes (e.g., stream power index and flow accumulation) were less important predictors in the RF approach. In contrast, in the regression kriging approach where we applied statistically significant environmental predictors, soil types, land cover types, stream power index, and sediment transport index were the most important predictors of surface SOC stocks. Likewise, the bedrock geology type was the least important but a statistically significant predictor of surface SOC stocks in the regression kriging approach.

### Comparison of Prediction Accuracy in Different Approaches

**FIGURE 7:**
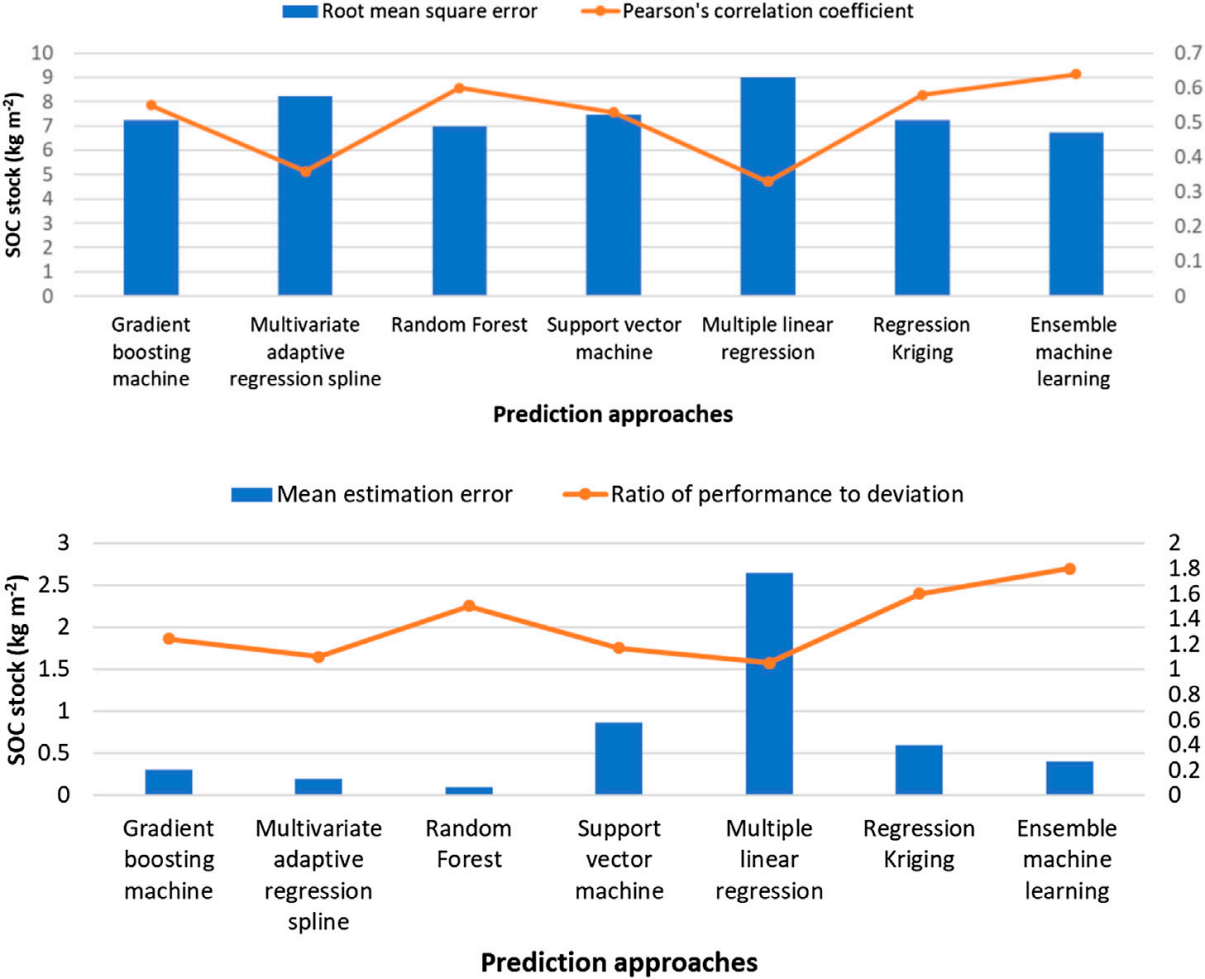
Average validation indices calculated for four different randomly selected calibration and validation datasets (70/30, 75/25, 85/15, 90/10) across the study area.

The predicted SOC stocks using an ensemble median of machine learning approaches showed lowest prediction errors (r = 0.64 and RMSE = 6.75 kg m^−2^; Figure 7) among all the spatial prediction approaches we evaluated. MLR and MARS produced highest prediction errors (r = 0.33 and 0.36, and RMSE = 9.0 and 8.25 kg m^−2^) among all the approaches we evaluated. Similarly, RF and regression kriging produced comparable prediction accuracies (comparable r and RMSE values; Figure 7). The average error of prediction was largest in the MLR approach followed by the MARS approach. On average, all prediction approaches showed positive bias (positive MEE values) and over predicted surface SOC stocks. The MLR and SVM techniques showed largest biases in SOC stock predictions, and RF showed smallest bias among all the prediction approaches. The RPD results showed that the SOC stock predictions from the ensemble median of machine learning approaches had an RPD value of 1.8. This moderate predictive accuracy is higher than any individual approach we applied (Chang and Laird, 2002; Viscarra Rossel and Webster, 2012). Other individual spatial prediction approaches showed lower predictive ability.

## Discussion

We compared multiple spatial prediction techniques to predict the surface SOC stocks of the northern circumpolar region. Calculated validation errors showed comparable prediction accuracies of GBM, RF, and regression kriging approaches. Prediction errors obtained from the ensemble median predictions of machine learning approaches were lowest in comparison to the regression kriging and other individual machine learning approaches. Our results show the distribution of the magnitude of uncertainty in SOC stocks across the northern circumpolar region, which can be used to guide future sampling efforts in order to reduce the modeled climate carbon feedback predictions.

Using a different thematic upscaling approach, Tarnocai et al. (2009) and Hugelius et al. (2014) predicted 191 and 217 Pg C in the surface soils of the northern circumpolar region. Our estimate of total SOC stocks ($218_{-26}^{+22}$ Pg C) is consistent with these previous estimates of the northern circumpolar region surface SOC stocks. However, our results showed different spatial distribution of SOC stocks across the study area. Our results showed 54% of SOC stocks reside in the continuous permafrost region, compared with 58% in Tarnocai et al. (2009). We report 18% SOC stocks in the discontinuous permafrost region, compared with 13% estimate in Tarnocai et al. (2009). Both our and Tarnocai et al. (2009) SOC stock estimates were similar in the sporadic permafrost region (14% SOC stocks). Our estimates showed similar SOC stocks in the isolated permafrost region as reported in Tarnocai et al. (2009) (<2%). Hugelius et al. (2014) used a different physiographic categorization to describe the spatial distribution of SOC stocks, and do not provide distributions of SOC stocks in different types of permafrost regions. In contrast to these previous estimates of surface SOC stocks, our approach provides greater spatial details and captures a larger range in predicted SOC stocks, primarily due to the larger number of field observations available to us and different geospatial approaches we used. Our ensemble median machine learning approach also elucidated impacts of different environmental variables representing various soil-forming factors, however, while both Tarnocai et al. (2009) and Hugelius et al. (2014) showed the impact of soil types only.

Areas with high uncertainty (>50%) in predicted SOC stocks have higher elevation and slope positions. Areas with low predicted uncertainty in SOC stocks have lower elevation and slope positions (Table 2). On average, areas with high uncertainty in SOC stocks receive lower precipitation and have drier soils. We note that these areas also have very few field observations (∼2% of total samples). The areas with medium and low uncertainty ranges have 37 and 61% of the observational samples, respectively. Our results of uncertainty distributions are consistent with findings of Shelef et al. (2017), who also reported that northern circumpolar region areas with high SOC stock uncertainty are areas with hillslope topography. The environmental characteristics and sample numbers of areas with different uncertainty ranges in the predicted surface SOC stocks of the northern circumpolar region are provided in Table 2. In order to reduce the existing uncertainty in surface SOC stocks, future sampling efforts should focus in the areas represented by green and purple colors in the Figure 5 (Table 2).

**TABLE 2:** Average values of environmental factors and number of samples in areas with different uncertainty ranges in the predicted surface SOC stocks of the northern circumpolar region.

Environmental factors	Uncertainty ranges		
	Low (>20%)	Medium (20–9%)	High (>50%)
Elevation (m)	408.0 (0–4,707)	575.0 (0–4,711)	687.0 (0–4,086)
Slope (°)	4.0 (0.5–71)	5.5 (0.5–72.2)	7.0 (0.5–64.6)
Temperature (^o^C)	−7.7 (−25.5–9.5)	−8.0 (−25.65–9.7)	−8.5 (−25.6–9.5)
Precipitation (mm)	407.0 (54–1751)	415.0 (54–2044)	395.0 (57–2,861)
Soil wetness index	9.5 (4.9–13)	9.0 (4.4–13)	8.0 (4.8–12.7)
Sample number	1,006	620	34

Values in parentheses show the range of environmental factors.

In this study, we used data of environmental factors that provided spatially-explicit information of major soil-forming factors across the study area. The impact of these environmental factors on soil formation is well documented in soil science literature (Jenny, 1941; McBratney et al., 2003; Mishra and Riley, 2012; Vitharana et al., 2017). For example, average annual precipitation and temperature data provided information of the average climate of the study area. Land cover types provided information on biota properties. Various topographic attributes that we used in this study provided information on relief. Soil types and bedrock geology types data provided information about different kinds of parent materials that impacted soil properties. In addition, in the permafrost domain, soil formation is also governed by cryopedogenic processes where the role of cold temperatures and ice formation are important for SOC stock accumulation and decomposition (Bockheim, 2007; Ping et al., 2013; Ping et al., 2015).

The regression kriging approach combines both environmental correlation and spatial autocorrelation to predict SOC stocks. As a result, regression kriging usually produces lower prediction errors in comparison to other approaches (Hengl et al., 2007; Mishra et al., 2012; Minasny et al., 2013). Our results suggest that some machine learning approaches, such as GBM and RF, that capture the non-linear relations between environmental controllers and SOC stocks, can produce similar prediction accuracy to that of regression kriging. We note that not all the machine learning approaches (for example, SVM and MARS) produced comparable prediction accuracy as was obtained from the regression kriging approach. However, the ensemble median prediction of machine learning approaches convincingly decreased the prediction errors and resulted in the most accurate predictions of surface SOC stocks but did not allow attribution of importance of individual environmental factors. Future studies should focus on 1) deriving the non-linear relationships between soil properties and the environmental factors and (2) incorporating the spatial autocorrelation function into machine learning approaches to achieve greater prediction accuracies.

## Summary

We compared multiple spatial prediction approaches to predict the surface SOC stocks of the northern circumpolar permafrost region. Using a larger number of samples than previously available, we compared the prediction accuracy of the regression kriging approach with four machine learning approaches. We found that SOC stock predictions from two machine learning approaches (GBM and RF) and regression kriging have comparable prediction accuracies. Prediction errors obtained from the ensemble median predictions of machine learning approaches were lowest in comparison to the regression kriging and other individual machine learning approaches. The number and importance of environmental predictors differed among different prediction approaches. Among machine learning approaches, temperature, latitude, land cover types, slope, and elevation had higher impacts on the predicted spatial variation of surface SOC stocks. Soil types were also important predictors in the regression kriging approach. We found an inverse spatial relationship between the magnitude of SOC stocks and its uncertainty. The uncertainty in surface SOC stocks was less than 20% in about half of the study area. Areas with high uncertainty (>50% uncertainty) in predicted SOC stocks were observed in small patches in Southern Alaska and Iceland, and in larger areas of Southern and Western Russian permafrost region. Because different machine learning approaches make use of different environmental predictors, the ensemble approach provides greater spatial details, and would probably provide improved estimates of SOC changes as it captures the non-linear relations between SOC stocks and its environmental predictors.

## Data Availability Statement

The data used in this study are available in supporting information section. Additional data and codes can be requested from the lead author.

## Author Contributions

UM and SG designed research, conducted analysis and wrote the manuscript. WR and FH assisted in discussion of the results and preparation of manuscript.

## Funding

This study was supported by the Director, Office of Science, Office of Biological and Environmental Research of the U.S. Department of Energy under Argonne National Laboratory contract No. DE-AC02-06CH11357. Efforts of WR were supported by the RUBISCO Scientific Focus Area in the Regional Global Climate Modeling Program by the Director, Office of Science, Office of Biological and Environmental Research, of the U.S. Department of Energy under contract DE-AC02-05CH11231 to Berkeley Lab.

## Disclaimer

This paper describes objective technical results and analysis. Any subjective views or opinions that might be expressed in the paper do not necessarily represent the views of the U.S. Department of Energy or the United States Government.

## Conflict of Interest

The authors declare that the research was conducted in the absence of any commercial or financial relationships that could be construed as a potential conflict of interest.
